# Impaired vascular function with age and RhoGTPase

**DOI:** 10.18632/aging.102739

**Published:** 2020-01-06

**Authors:** Edward Dempsey, Derek Strassheim, Vijaya Karoor

**Affiliations:** 1 Cardiovascular Pulmonary Research Laboratory, Department of Medicine, University of Colorado Denver, Aurora, CO 80045, USA

**Keywords:** arterial stiffening, RhoGTPases, inflammation, oxidative stress, endothelial cell, vascular smooth muscle cell

Aging increases oxidative stress, inflammation, cellular senescence, and decreases autophagy in the vasculature [1]. Vascular remodeling and arterial stiffening of large conduit arteries occur due to an increase in collagen deposition and elastin fragmentation resulting in a decrease in compliance. In older arteries, increased activation of RhoA leads to an increase in basal tone in the vasculature. RhoA-ROCK signaling is vital in the expression of contractile proteins in vascular smooth muscle cells. However, sustained RhoGTPase activation leads dedifferentiation of SMC and vascular remodeling [2]. Protein kinase G regulates vascular tone by inhibition of RhoA by phosphorylation. PKG levels decrease with age, which could lead to enhanced vasoconstriction of arteries [1]. Vasoconstriction and impaired vasodilation reduce lumen diameter, increase wall-to-lumen ratio, and inward remodeling of small arteries.

Arterial calcification also contributes to vessel stiffness, and is associated with an increase in the transcription factor Runx2, and type I collagen expression in smooth muscle cells. RhoA suppresses the expression of Klotho, a membrane protein associated with longevity [1]. In the Klotho (-/-) aging mouse model, osteoblast-like cells express RUNX-2 in the calcified media, which was attenuated by inhibition of the RhoA-ROCK pathway [3]. Vascular protection due to statins may include their ability to increase Klotho expression. Aging dependent increases in extracellular matrix lead to arterial stiffening in mouse models, which is decreased by inhibitors of RhoA-ROCK signaling.

Endothelial dysfunction with age occurs due to increases in vascular permeability, oxidative stress, and inflammation [1]. A decrease in eNOS increases endothelial permeability by increasing Rho activity. Aging increases endothelial cell arginase activity via RhoA, which competes with eNOS for the common substrate L-arginine leading to decreased eNOS-NO-vasodilation. Arginase levels are increased in cardiovascular complications of diabetes and hypertension. Studies in animal models indicate that increased ROCK activity causes uncoupling of eNOS, resulting in decreased NO availability and increased reactive nitrogen species and oxidative stress. In diabetic retinopathy, Rac1 activation by Nox2-ROS in the vasculature contributes to mitochondrial damage. In smooth muscle cells, ROS increases levels of cyclophilin A in a Rho-dependent manner [4]. Oxidative stress also enhances nuclear factor-κB activation and promotes proinflammatory cytokine secretion, leading to medial thickening.

An increase in senescent endothelial and smooth muscle cells occurs in cardiovascular diseases, including heart failure, diabetes, and atherosclerosis. Aging causes telomere shortening, cell senescence, and inflammation in the vasculature [1]. In endothelial cells, the senescence marker protein-30 (SMP30) confers protection and declines with age in a process that appears to be driven by the RhoA-ROCK axis [5]. Paraoxonase 1 protects against ROS, vascular aging, and senescence in endothelial cells. Paraoxonase 1 knockdown decreases levels of RhoGDI and increases levels of senescence marker β-galactosidase [6]. SIRT1, an HDAC, reduces endothelial senescence by increasing eNOS and Foxo1 and decreasing RhoA-ROCK signaling. Increasing SIRT1 levels and maybe a therapeutic strategy in age-related dysfunction of the vasculature.

Recent studies have emphasized the role of autophagy as a critical regulator of the aging process, primarily through the removal of damaged mitochondria [1]. Genetic knockout of ROCK1 in mice affected autophagosome formation and impaired autophagy [7]. In spontaneously hypertensive rats, treatment with autophagy activator-trehalose increased vasodilator responses to acetylcholine and decreased ROCK activity and arterial stiffness [8]. RhoA represses mTOR signaling, which has a role both in autophagy and vascular aging.

Age-related decline in function is a physiological phenomenon occurring in all organ systems. However, cardiovascular risk factors, such as hypertension, smoking, hyperlipidemia, obesity or diabetes mellitus, enhance the decline of vascular function. The vascular protective effects of statins and DPPIV inhibitors include their ability to reduce RhoA/activity. Population-based studies show that arterial stiffening precedes vascular dysfunction in chronic disease and RhoGTPases may have a role. RhoGTPases modulate various pathways that contribute to aging. Therefore studying effect of age on RhoGTPases and their regulators in preclinical models of chronic disease may reveal whether they can be targets in reversing aging-induced vascular dysfunction.
